# Intracellular HMGB1: defender of client proteins and cell fate

**DOI:** 10.18632/oncotarget.3836

**Published:** 2015-04-15

**Authors:** Jeannette Messer, Eugene Chang

**Affiliations:** Department of Medicine, University of Chicago, Chicago, IL, USA

Inflammatory and infectious diseases are often characterized by large amounts of epithelial cell death. This is particularly true for the inflammatory bowel diseases (IBD) of the gastrointestinal tract. Under normal homeostatic conditions, epithelial cells lining the intestines (IEC) undergo an orderly progression from undifferentiated, self-renewing stem cells at the base of crypts to differentiated absorptive and secretory cells in the villi. Cell survival and cell death cues are highly regulated in this process allowing for rapid proliferation in the depths of the crypt and programmed cell death at the villus tips. However, during IBD, IEC death programs become disordered and are activated on a large scale, leading to loss of the normal digestive and anti-microbial barrier functions of the intestinal epithelium. Although IEC death has historically been attributed to adaptive immune system activation and mucosal infiltration by cytotoxic T cells; strategies targeted at suppressing adaptive immune responses have had limited success in preventing IEC death and fully restoring the gastrointestinal mucosal barrier. This suggests that alterations in IEC themselves during inflammation are likely an important contributor to the increased death of this cell population in IBD.

Among the agents implicated in adaptive immune damage to the intestinal epithelium is high mobility group box 1 (HMGB1). Extracellular HMGB1 is thought to act predominantly as a late, pro-inflammatory cytokine during inflammatory diseases. This is due to evidence that drugs that “prevent HMGB1 release” or “HMGB1 neutralizing antibody” ameliorate disease in murine models of inflammatory disease and that increased levels of HMGB1 are found in the blood of patients with active inflammatory diseases. HMGB1 is also found inside cells where it undergoes a shift from the nucleus to the cell cytosol during inflammation. The role of intracellular HMGB1 in inflammatory disease has been largely ignored, partially due to a lack of physiologic mouse models since global HMGB1 deficiency is lethal in mice. Our recent work published in the Journal of Clinical Investigation [1] explored the role of intracellular HMGB1 in inflammation using samples of intestinal tissue derived from IBD patients and mice conditionally deficient in IEC HMGB1. In these studies, patients with active IBD were found to have decreased levels of HMGB1 protein in their gastrointestinal mucosa, with a shift in the IEC intracellular localization to the cell cytosol. Concurrently, mice deficient in IEC HMGB1 developed worse colitis in the dextran sodium sulfate and IL-10^−/−^ disease models. We also examined HMGB1 deficient primary IEC grown *in vitro* as organoids. In these studies, we found that HMGB1 has a cell-intrinsic role in cell fate decisions through regulation of calpain-mediated cleavage of the autophagy proteins beclin-1 and Atg5. Beclin-1 and Atg5 are unique among the autophagy proteins in that they can be cleaved to produce pro-apoptic protein fragments. Thus, HMGB1 controls the switch between these two functions of beclin-1 and Atg5 and the intracellular availability of HMGB1 determines whether cells undergo autophagy and survive inflammation or activate cell death programs. Autophagy is generally considered to be a pro-survival process within cells. During stress it encapsulates damaged cellular contents or microbial invaders into double membrane vesicles and then transports these vesicles to lysosomes to destroy the vesicle contents. Autophagy failure has been linked to cell death by a variety of mechanisms, but our results demonstrate that autophagy failure is entwined with de novo generation of the pro-apoptotic fragments of beclin-1 and Atg5 during calpain-mediated inflammation. This finding has important implications for many different inflammatory and infectious diseases since high levels of calpain activation have been identified in cardiomyopathy, type 2 diabetes, ischemia-reperfusion, microbial infections, and cancers.

The idea that intracellular HMGB1 has a pivotal role in cell fate during inflammation is further supported by other recent studies in mice conditionally deficient in HMGB1 in a variety of cell types. Specifically, studies using our HMGB1 floxed mice have shown that conditional ablation of HMGB1 in pancreatic cells worsens disease in L-arginine and cerulean-induced pancreatitis and that deficiency of HMGB1 in hepatocytes worsens disease secondary to ischemia and reperfusion [2,3]. Studies using a second, independently generated, floxed HMGB1 mouse line have shown that monocytic cell deletion of HMGB1 worsens LPS-induced endotoxemia, without changing blood levels of HMGB1 [4]. Collectively, these data suggest that extracellular HMGB1 has a less dominant role in the pathophysiology of inflammatory diseases such as IBD than previously thought, and that the original findings of increased extracellular HMGB1 during inflammation should be revisited in the emerging context of intracellular HMGB1 deficiency in human disease.

Ultimately, the severity of many human infectious and inflammatory diseases is related to the severity of cell death within the affected tissue. Cell death may compromise essential organ functions, release pro-inflammatory mediators, or allow microbes access to normally sterile sites within the organism. Our data and that of others examining the role of HMGB1 functions using physiologically relevant models shows that intracellular HMGB1 is a key determinant of cell death during inflammation. Therefore, intracellular HMGB1 is likely to be the clinically relevant pool of the protein in at least a subset of diseases and is worthy of increased scrutiny and investigation. Understanding the role of intracellular HMGB1 in human disease is particularly important since therapeutic strategies targeting extracellular HMGB1 have been proposed to treat inflammatory diseases. Perhaps a better strategy would be selective manipulation of this protein within specific cell types during inflammation.

## References

[1] Zhu X. (2015). J. Clin. Invest.

[2] Huang H. (2014). Hepatology.

[3] Kang R. (2014). Gastroenterology.

[4] Yanai H. (2013). Proc. Natl. Acad. Sci. U. S. A.

